# VEGF‐A_165_b protects against proteinuria in a mouse model with progressive depletion of all endogenous VEGF‐A splice isoforms from the kidney

**DOI:** 10.1113/JP274481

**Published:** 2017-07-03

**Authors:** Megan Stevens, Christopher R. Neal, Andrew H. J. Salmon, David O. Bates, Steven J. Harper, Sebastian Oltean

**Affiliations:** ^1^ School of Physiology, Pharmacology and Neurosciences University of Bristol UK; ^2^ Bristol Renal, School of Clinical Sciences University of Bristol Bristol UK; ^3^ Cancer Biology, Division of Cancer and Stem Cells, School of Medicine University of Nottingham Nottingham UK; ^4^ Present address: Institute of Biomedical & Clinical Sciences, University of Exeter Medical School Exeter UK

**Keywords:** alternative splicing, reno‐protection, vascular endothelial growth factor

## Abstract

**Key points:**

- Progressive depletion of all vascular endothelial growth factor A (VEGF‐A) splice isoforms from the kidney results in proteinuria and increased glomerular water permeability, which are both rescued by over‐expression of VEGF‐A_165_b only.
- VEGF‐A_165_b rescues the increase in glomerular basement membrane and podocyte slit width, as well as the decrease in sub‐podocyte space coverage, produced by VEGF‐A depletion.
- VEGF‐A_165_b restores the expression of platelet endothelial cell adhesion molecule in glomerular endothelial cells and glomerular capillary circumference.
- VEGF‐A_165_b has opposite effects to VEGF‐A_165_ on the expression of genes involved in endothelial cell migration and proliferation.

**Abstract:**

Chronic kidney disease is strongly associated with a decrease in the expression of vascular endothelial growth factor A (VEGF‐A). However, little is known about the contribution of VEGF‐A splice isoforms to kidney physiology and pathology. Previous studies suggest that the splice isoform VEGF‐A_165_b (resulting from alternative usage of a 3′ splice site in the terminal exon) is protective for kidney function. In the present study, we show, in a quad‐transgenic model, that over‐expression of VEGF‐A_165_b alone is sufficient to rescue the increase in proteinuria, as well as glomerular water permeability, in the context of progressive depletion of all VEGF‐A isoforms from the podocytes. Ultrastructural studies show that the glomerular basement membrane is thickened, podocyte slit width is increased and sub‐podocyte space coverage is reduced when VEGF‐A is depleted, all of which are rescued in VEGF‐A_165_b over‐expressors. VEGF‐A_165_b restores the expression of platelet endothelial cell adhesion molecule‐1 in glomerular endothelial cells and glomerular capillary circumference. Mechanistically, it increases VEGF receptor 2 expression both *in vivo* and *in vitro* and down‐regulates genes involved in migration and proliferation of endothelial cells, otherwise up‐regulated by the canonical isoform VEGF‐A_165_. The results of the present study indicate that manipulation of VEGF‐A splice isoforms could be a novel therapeutic avenue in chronic glomerular disease.

## Key points

- Progressive depletion of all vascular endothelial growth factor A (VEGF‐A) splice isoforms from the kidney results in proteinuria and increased glomerular water permeability, which are both rescued by over‐expression of VEGF‐A_165_b only.
- VEGF‐A_165_b rescues the increase in glomerular basement membrane and podocyte slit width, as well as the decrease in sub‐podocyte space coverage, produced by VEGF‐A depletion.
- VEGF‐A_165_b restores the expression of platelet endothelial cell adhesion molecule in glomerular endothelial cells and glomerular capillary circumference.
- VEGF‐A_165_b has opposite effects to VEGF‐A_165_ on the expression of genes involved in endothelial cell migration and proliferation.

**Abbreviations**
ACTG1: actin gamma‐1
ciGEnCs: conditionally immortalized glomerular endothelial cells
CKD: chronic kidney disease
DEPC: diethylpyrocarbonate
GBM: glomerular basement membrane
GFR: glomerular filtration rate
GSI: glomerulosclerosis index
ISH: *in situ* hybridization
KD: knockdown
KO: knockout
L_p_A/V_i_: glomerular ultrafiltration coefficient normalized to glomerular area
PAS: periodic acid‐Schiff
PECAM: platelet endothelial cell adhesion molecule
PFA: paraformaldehyde
SHC1: Src homology 2 domain containing transforming protein 1
SPS: sub‐podocyte space
SSC: saline sodium citrate
TBS: Tris‐buffered saline
uACR: urinary albumin creatinine ratio
VEGF‐A: vascular endothelial growth factor A
VEGFR: vascular endothelial growth factor receptor
WT: wild‐type

## Introduction

Chronic kidney disease (CKD) is a major health problem worldwide. In the USA, it is estimated that ∼13% of the general population has CKD stages 1–4 (Snyder *et al*. 2009) and, in Europe, the prevalence is higher at 16% (Meguid El Nahas & Bello, 2005). The renal replacement population inevitably consumes a disproportionate amount of health resources and is associated with a poor prognosis, with a 5 year survival rate of close to 50% for dialysis patients (https://www.renalreg.org/data/). In large part, this is contributed to by the association of proteinuria and CKD with increased risk of cardiovascular disease and mortality (Go *et al*. 2004; Allison, 2010; Matsushita *et al*. 2010). Therefore, it is critical to elucidate molecular determinants of glomerular dysfunction and CKD and find new ways to manipulate them for therapeutic benefit.

Abnormal expression of vascular endothelial growth factor A (VEGF‐A) in the kidney has been reported in many renal pathologies, such as diabetic nephropathy, several glomerulopathies (e.g. membranous glomerulopathy, focal‐segmental glomerulosclerosis) and acute and chronic transplant rejection (Schrijvers *et al*. 2004). Interestingly, a recent study that analysed expression networks in 157 biopsies of patients with CKD resulting from various causes identified VEGF‐A as one of the genes significantly correlated with renal function, both in glomerular and tubulointerstitial compartments (Martini *et al*. 2014). Moreover, with the advent of anti‐VEGF/VEGF receptor (VEGFR)2 therapies in oncology, several side effects including renal complications have been reported, with proteinuria resulting from thrombotic microangiopathy, minimal change nephrotic syndrome and focal‐segmental glomerulosclerosis (Chen & Cleck, 2009; Ollero & Sahali, 2014). Therefore, there is a clear physiological role of VEGF‐A in the kidney, which is affected in various pathologies. This suggests that restoring the function of VEGF‐A could be therapeutically useful.

Several studies have reported that knockout (KO) or knockdown (KD) of VEGF‐A from the kidney in transgenic mouse models has various deleterious effects depending on the age at which the KO/KD is induced or whether they are challenged on a diabetic background (Eremina *et al*. 2008; Sivaskandarajah *et al*. 2012; Veron *et al*. 2012). However, these studies have not until now taken into account the effect of VEGF‐A splice isoforms, such as VEGF‐A_165_b, resulting from an alternative 3′ splice site in the terminal exon. VEGF‐A_165_b has been shown to be a partial agonist/antagonist of VEGFR2 and to be protective of kidney function (Bates *et al*. 2002; Bevan *et al*. 2008; Harper & Bates, 2008; Qiu *et al*. 2010; Oltean *et al*. 2012; Oltean *et al*. 2015) *in vivo* and protective for podocyte function *in vitro* (Bevan *et al*. 2008); however, it does not affect normal kidney function when over‐expressed in a podocyte‐specific manner (Qiu *et al*. 2010) and it is able to counteract the pro‐permeability function of the VEGF‐A_165_ splice isoform *in vivo* (Oltean *et al*. 2012).

Given the pathologies associated with VEGF‐A inhibition and the strong association of decreased glomerular filtration rate (GFR) with low VEGF‐A expression, we have addressed the hypothesis that a simplified injury model of progressive cell‐specific inducible depletion of VEGF‐A from the kidney can be rescued by over‐expressing only the VEGF‐A_165_b isoform. This the first study to determine the effects of podocyte VEGF‐A_165_b over‐expression when all other isoforms of VEGF‐A are depleted. We find that a KD of podocyte VEGF‐A expression in adult mice results in microalbuminuria and increased glomerular permeability, without any evidence of glomerulosclerosis/fibrosis. Furthermore, we find that VEGF‐A_165_b alone is sufficient to restore normal kidney function, and we go on to describe its mechanism of action *in vivo* and how this differs from that of VEGF‐A_165_a in conditionally immortalized glomerular endothelial cells (ciGEnCs) in culture.

## Methods

### Ethical approval

All experiments were conducted in accordance with UK legislation and local ethical committee approval, as well as in accordance with the principles and regulations as described by Grundy (2015). Animal studies were approved by University of Bristol research ethics committee.

### Materials

All materials were obtained from Sigma (Poole, UK) unless otherwise stated.

### Mouse models

Transgenic mice were obtained from in‐house colonies. Mice were entered into studies at 8 weeks of age and wild‐type (WT) littermate controls were used for comparison. Details for the pod‐rtTA and Neph‐VEGF‐A_165_b mice are provided elsewhere (Qiu *et al*. 2010; Oltean *et al*. 2012). Offspring were genotyped by PCR analysis of an ear notch for each of the four transgenes: Pod‐rtTA, Tet‐O‐Cre, LoxP VEGF‐A and Neph‐VEGF‐A_165_b. Excision of exon 3 of VEGF‐A in the podocytes was induced by adding doxycycline (5 mg ml^−1^) to light‐protected drinking water containing 5% w/v sucrose. Doxycyline was administered for up to 14 weeks to inducible podocyte‐specific VEGF‐A‐KO mice, VEGF‐A‐KO mice × Neph‐VEGF‐A_165_b mice and WT littermate controls (+:+:Pod‐rtTA:LoxP‐VEGF). VEGF‐A_165_b over‐expression was constitutive in podocytes in mice containing the Neph‐VEGF‐A_165_b transgene. Four to 12 mice were used per group over two experiments. Mice were culled at the end of the experiment via Schedule 1 killing (cervical dislocation).

### *In situ* hybridization (ISH)

ISH was carried out on 5 μm thick, 4% paraformaldehyde‐fixed kidney cortex sections using a digoxigenin‐labelled VEGF‐A_164_ riboprobe on VEGF‐A‐KO and WT littermate controls 4, 10 and 14 weeks after induction with doxycycline. ISH for VEGF‐A_164_ mRNA was used to determine the level of VEGF‐A KD in the glomerulus. The VEGF‐A_164_ cDNA cloned into a Bluescript II ks plasmid was a kind gift from Vera Eremina (University of Toronto, Toronto, Canada). Digoxigen‐labelled probes were prepared in accordance with the manufacturer's instructions (Roche, Mannheim, Germany). Sections were air‐dried overnight at room temperature and then treated with 15 μg ml^−1^ proteinase K (Roche) in diethylpyrocarbonate (DEPC)‐treated PBS at 37°C for 5 min.

Sections were fixed in 4% paraformaldehyde (PFA) in DEPC‐treated PBS at room temperature for 7 min, and then washed firstly in DEPC‐treated PBS and then in 2 × saline sodium citrate (SSC) buffer. Sections were pre‐hybridized in preheated (60°C) hybridization buffer (50% formamide, 5 × SSC, 0.5% Chaps; 0.1% Triton X‐100, 5 μg ml^−1^ yeast tRNA; 5 μg ml^−1^ heparin, 5 mm EDTA, 2% blocking powder) in a 50% formamide/5 × SSC humidified chamber at 60°C for 1 h. The pre‐hybridization buffer was removed and replaced with hybridization buffer containing the probe at 1 μg ml^−1^ and incubated overnight at 60°C. The next day, slides were washed in a 50% formamide/0.2 × SSC solution at 60°C for 15 min. Sections were then incubated in NT blocking buffer (Roche) for 30 min before incubating in primary antibody: sheep anti‐digoxigenin AP Fab fragments (Roche) diluted 1:2000 in NT blocking buffer. Primary antibody was left on for 2 h at room temperature. Slides were washed in Tris‐buffered saline (TBS)‐Tween (0.1%) with 2 mm levamisole for 30 min and then in APB buffer (1 × TBS‐Tween (0.1%) with 0.2 m levamisole) with agitation for 15 min. Slides were then coated in BM purple colour substrate (Roche) and placed in a dark humid chamber at room temperature overnight or until colour developed. Fotr to eight mice were used from each group, with fiuve to seven glomeruli analysed per mouse. Staining was repeated three times per mouse and averages were taken.

### RT‐PCR

RNA was extracted with Trizol (Invitrogen, Carlsbad, CA, USA) from WT, VEGF‐A‐KO and VEGF‐A‐KO × Neph‐VEGF‐A_165_b kidney cortex and then *DNase*I (Promega, Madison, WI, USA) digested to prevent genomic DNA contamination. Next, 2 μg of DNase‐treated RNA was reverse transcribed into cDNA with avian myeloblastosis virus reverse transcriptase in accordance with the manufacturer's instructions (Promega). Both cDNA and RNA [(RT^−^) control] were subjected to PCR as described previously (Qiu *et al*. 2010) to indicate VEGF‐A_165_b transgene expression with a band at 199 bp. Three mice per group were studied and the experiment was repeated three times.

### Functional phenotype

Urine was collected by housing mice in metabolic cages for 6–12 h. Urine was first collected before doxycycline administration began for baseline readings, and then every 2 weeks up until mice were killed 14 weeks after induction began. The urinary albumin creatinine ratio was used as a measure of urinary protein loss. Albumin was quantified with an albumin enzyme‐linked immunosorbent assay (Bethyl Laboratories, Inc., Montgomery, TX, USA) and creatinine was quantified using an enzymatic spectrophotometric assay (Konelab T‐Sereis 981845; Thermo Fisher Scientific, Waltham, MA, USA). Four to 12 mice were used from each group, with assays being repeated in triplicate. Upon culling mice, blood was collected for plasma creatinine analysis. Kidneys were immediately removed and a small, finely diced, section of cortex was fixed in 2.5% gluteraldehyde in 0.1 m cacodylate buffer for electron microscopy. Sections of kidney cortex were snap frozen in liquid nitrogen and stored at −80°C for subsequent RNA and protein analysis, and also in embedding medium for sectioning and staining. The remaining kidney was used to harvest glomeruli using a standard sieving technique for glomerular ultrafiltration coefficient normalized to glomerular area (L_p_A/V_i_) experiments using an oncometric assay described previously (Salmon *et al*. 2006). In one set of experiments, glomeruli from WT and Neph‐VEGF‐A_165_b mice were incubated for 1 h with 100 nm PTK787 at 4°C before measuring glomerular L_p_A/V_i_. Four to nine mice were used from each group, with four to five glomeruli being analysed per mouse.

### Structural and ultrastructural phenotype

Regarding structural and ultrastructural phenotype, 4% paraformaldehyde‐fixed kidney cortex sections were stained with a periodic acid‐Schiff (PAS) and Masson's trichrome blue stain to determine the structural glomerular phenotype. PAS stained glomeruli were blindly scored using the widely‐used glomerulosclerosis index (GSI) (1 = normal; 2 = 25% sclerosis; 3 = 50% sclerosis; 4 = < 75% sclerosis). Masson's trichrome stained sections were blindly assessed for percentage area of glomeruli stained blue (collagen).

The ultrastructural phenotype was determined using 2.5% glutaraldehyde‐fixed diced kidney cortex, which was postfixed in 1% osmium tetroxide and embedded in Araldite (Agar Scientific, Stansted, UK). Sections of 50–100 nm thickness were stained with 3% (aqueous) uranyl acetate and Reynolds’ lead citrate solution. From the images taken, detailed measurements of the glomerular filtration barrier were taken by a blinded experimenter at random points using Photoshop (Adobe Systems Inc., San Jose, CA, USA). These included glomerular basement membrane (GBM) thickness, endothelial fenestration number, average podocyte foot process width, average podocyte slit width and percentage sub‐podocyte space (SPS) coverage. Three mice were used per group.

### Immunofluorescence

Kidney samples for immunofluorescence studies were snap frozen in OCT embedding medium (Invitrogen). Then, 5 μm sections were mounted on to glass slides and fixed for 15 min with 4% PFA. Primary antibodies used were anti‐VEGFR2 (dilution 1:50; Cell Signaling Technology, Beverly, MA, USA), anti‐nephrin (dilution 1:250; Acris GmbH, Herford, Germany), anti‐podocin (dilution 1:1:500; Sigma, St Louis, MO, USA) and anti‐platelet‐endothelial cell adhesion molecule (PECAM)‐1 (dilution 1:1:500; BD Biosciences, San Jose, CA, USA). The appropriate Alexa‐Fluor secondary antibody was used and sections were imaged on a fluorescence microscope. Staining was normalized to glomerular area to calculate the percentage glomerular area covered by staining. Z stack confocal imaging was used to access co‐localization. Three mice were assessed per group, over three experiments. A minimum of 36 glomeruli were analysed per mouse.

### Cell culture studies

ciGEnCs (Satchell *et al*. 2006) were differentiated at 80% confluence with EBM2 media (Lonza Inc., Allendale, NJ, USA). Prior to treatment, cells were serum starved for 6 h before the addition of vehicle (PBS) 1 nm VEGF‐A_165_, 1 nm VEGF‐A_165_b, or a combination of both. Treatments lasted for 0.25, 0.5, 1, 2, 24 and 48 h. Protein was then extracted using a NP40 lysis buffer and RNA was extracted using Trizol (both from Invitrogen). Treatments were always carried out in triplicate with the relative controls. Experiments were repeated three times.

### Western blotting

Western blotting was performed on protein extracted from sieved glomeruli or kidney cortex and on protein extracted from treated ciGEnCs. Samples were run on mini‐PROTEAN® TGX Stain Free™ pre‐cast gels (4–15%; Bio‐Rad, Hercules, CA, USA) and transferred onto polyvinylidene fluoride membranes. Membranes were blocked in 3% BSA in TBS‐Tween (0.1%) for 1 h at room temperature and probed using anti‐P‐Tyr (dilution 1:1000; 9411; Cell Signaling) (after immunoprecipitation for VEGFR2; 55B11; Cell Signaling), nephrin (dilution 1:1000; Acris GmbH), podocin (dilution 1:1000; Sigma), PECAM (dilution 1:1000; Santa Cruz Biotechnology, Santa Cruz, CA, USA), p‐Akt (dilution 1:1000; Cell Signaling), Akt (dilution 1:1000; Cell Signaling), p‐p42/44 MAPK/p‐ERK1/2 (dilution 1:1000; Cell Signaling), p‐42/44 MAPK/ERK1/2 (dilution 1:1000; Cell Signaling), VEGF‐A_165_b (dilution 1:500; R&D Systems, Minneapolis, MN, USA), VEGF‐A_164_ (dilution 1:1000; Abcam, Cambriudge, UK), actin gamma‐1 (ACTG1) (dilution 1:1000; Genetex, Irvine, CA, USA) or Src homology 2 domain containing transforming protein 1 (SHC1) (dilution 1:1000; R&D Systems) at 4°C overnight. The appropriate Licor fluorescent secondary antibodies were applied for 2 h at room temperature and membranes were imaged using a Licor Imaging System (Licor, Lincoln, NE, USA). Total protein was measured using a Gel‐Doc EZ (Bio‐Rad), allowing quantification of total protein from the membrane. Experiments were repeated three times with the relative controls on the same blot.

### PCR array and validation

RNA extracted from 1 h treated ciGEnCs was used to make cDNA using the RT‐PCR process described above. A human VEGF‐A pathway TaqMan fast array (Applied Biosciences, Foster City, CA, USA) was used to examine gene expression profiles from the cDNA. Forty‐six candidate genes were tested, plus two housekeeping control genes. The fold change in gene expression relative to control (ΔΔCt) was measured in cDNA from ciGEnCs treated for 1 h with either VEGF‐A_165_ or VEGF‐A_165_b. cDNA was then subjected to quantitative RT‐PCR using gene specific primers for candidate genes identified within the PCR array for validation. Cells were treated in triplicate over four experiments.

### Statistical analysis

To test our hypothesis that the podocyte‐specific over‐expression VEGF‐A_165_b plays a protective role in the inducible podocyte‐specific VEGF‐A KD model of glomerular disease, we performed statistical analysis using Prism (GraphPad Software Inc., San Diego, CA, USA). The appropriate statistical test for the data set was used the analyse the data, including Student's unpaired *t* test and one‐way ANOVA, incorporating the non‐parametric equivalents if the data did not fall under the normal distribution. All analysis was blinded by the researcher, restricting bias. Details of numbers of repeats can be found under each individual method. The researcher was kept blinded to the group during imaging and analysis.

## Results

### Albuminuria resulting from progressive depletion of VEGF‐A in kidneys of inducible transgenic KO mice is rescued by constitutive expression of the human VEGF‐A_165_b splice isoform

**Figure 1:**
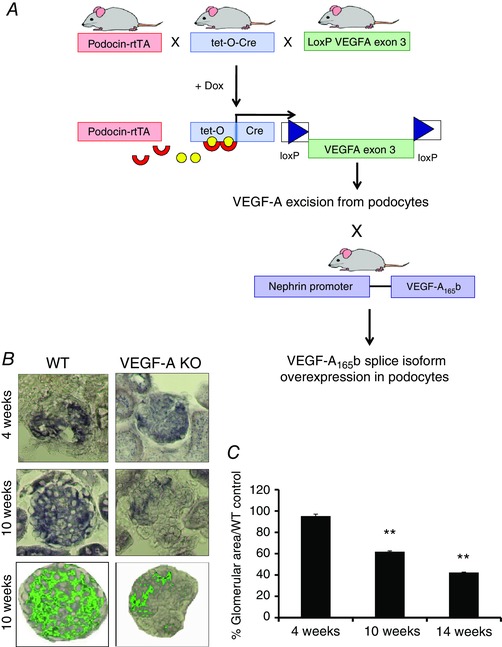
Generation of inducible podocyte‐specific VEGF‐A‐KO mice that over‐express VEGF‐A_165_b in the podocytes *A*, the inducible podocyte‐specific VEGF‐A‐KO mouse was generated from three starter colonies: Pod‐rtTA, Tet‐O‐Cre and LoxP VEGF‐A (LoxP sites flanking exon 3). In the absence of doxycycline (yellow circles), reverse tetracycline trans‐activator protein (rtTA, red crescents), under the control of the podocin promoter, is unable to bind to the Tet‐O‐Cre transgene at the tetracycline responsive element. When doxycycline is added to the drinking water, rtTA is able to bind and initiate transcription of Cre recombinase in podocytes, resulting in excision of exon 3 from VEGF‐A, and thus preventing the expression of protein VEGF‐A in the podocytes. This was crossed with the Neph‐VEGF‐A_165_b mouse to obtain the quadruple transgenic. *B*, mouse VEGF‐A_164_ mRNA levels were quantified by ISH on sections from the renal cortex. VEGF‐A_164_ mRNA expression was calculated (*C*) by normalizing the area stained purple (highlighted in green) to the total glomerular area, and expressing VEGF‐A_164_ expression as a percentage of glomerular area relative to the mean WT control group for each time point (*n* = 4–8 mice, 21–56 glomeruli. ^**^ *P* < 0.01; unpaired Student's *t* test for VEGF‐A‐KO *vs*. WT littermate controls for the same time point).

We investigated whether the VEGF‐A_165_b isoform is sufficient as a lone isoform for normal kidney function *in vivo*. Accordingly, we took advantage of a transgenic mouse model with over‐expression of human VEGF‐A_165_b under a nephrin promoter that we had characterized previously (Qiu *et al*. 2010), which has podocyte‐specific constitutive over‐expression of VEGF‐A_165_b in the glomerulus. This was crossed with a podocyte‐specific and inducible VEGF‐A‐KO to obtain quadruple transgenic mice (VEGF‐A‐KO × Neph‐VEGF‐A_165_b) (Fig. 1 *A*).

Induction of VEGF‐A‐KO from the kidney was started by adding doxycycline to the drinking water. At 4 weeks post‐induction, there was no difference in amount of VEGF‐A in the glomeruli of VEGF‐A‐KO mice compared to WT (+:+:pod‐rtTA:VEGF‐LoxP) controls, as quantified by *in situ* hybridization; however, significant and progressive depletion of VEGF‐A from the kidney was observed at 10 and 14 weeks post‐induction (Fig. 1 *B* and *C*) in the Pod‐rtTA:TetO‐Cre:VEGF‐LoxP triple transgenic mice. KD of VEGF‐A_164_ protein was also observed in the sieved glomeruli from VEGF‐A‐KO and VEGF‐A‐KO × Neph‐VEGF‐A_165_b mice via western blotting. VEGF‐A_165_b over‐expression was confirmed by RT‐PCR and western blotting and showed an average three‐fold increase in VEGF‐A_165_b expression in the kidney cortex of the quadruple transgenic mice, as described previously by Qiu *et al*. (2010) (data not shown).

**Figure 2:**
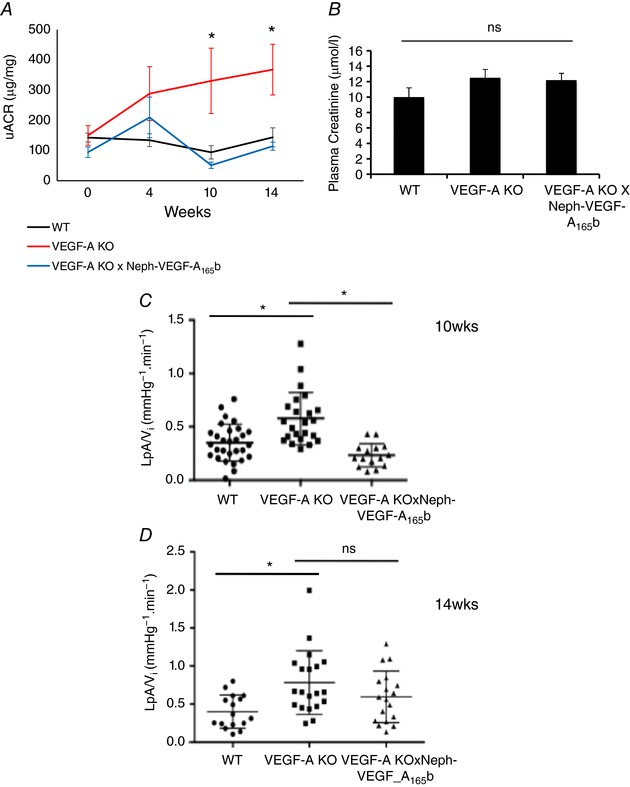
VEGF‐A‐KO mice develop albuminuria and have increased glomerular water permeability, which is rescued in VEGF‐A‐KO × Neph‐VEGF‐A_165_b mice *A*, uACR was determined from urine samples at 0, 4, 10 and 14 weeks after induction with doxycycline (*n* = 4–12 mice per time point). The uACR significantly increased in VEGF‐A‐KO mice at weeks 10 and 14 compared to WT littermate controls. This was fully rescued in VEGF‐A‐KO × Neph‐VEGF‐A_165_b mice (^*^ *P* < 0.05; two‐way ANOVA, correction for comparison between pairs). *B*, VEGF‐A‐KO mice did not develop increased plasma creatinine at the 14 week time point. VEGF‐A‐KO mice had a significantly increased glomerular water permeability (L_p_A/V_i_) at both 10 (*C*) and 14 (*D*) weeks after induction with doxycycline. This was fully rescued in the VEGF‐A‐KO × Neph‐VEGF‐A_165_b mice at the 10 week time point; however, there was no significant rescue by VEGF‐A_165_b at 14 weeks (*n* = 4–9 mice, 15–30 glomeruli; ^*^ *P* < 0.05; one‐way ANOVA, Bonferroni correction for comparison between pairs).

The urinary albumin:creatinine ratio (uACR) was significantly increased in VEGF‐A‐KO mice compared to WT littermates at 10 and 14 weeks post‐induction (WT; 93.9 ± 14.7, 143.8 ± 31.3, VEGF‐A‐KO; 330.7 ± 108.1, 367.9 ± 83.9, at 10 and 14 weeks, respectively, all units are μg mg^–1^) and was rescued by over‐expression of VEGF‐A_165_b under a nephrin promoter (VEGF‐A‐KO × Neph‐VEGF‐A_165_b; 51.1 ± 10.6, 114.2 ± 13.3, at 10 and 14 weeks, respectively, all units are μg mg^–1^) (*P* < 0.05; two‐way ANOVA) (Fig. 2 *A*). No significant increase in plasma creatinine was observed in VEGF‐A‐KO mice (Fig. 2 *B*). However, glomerular water permeability was increased in a manner similar to albuminuria in VEGF‐A‐KO mice, which was rescued by constitutive expression of VEGF‐A_165_b at 10 weeks post‐induction (Fig. 2 *C*) but not at 14 weeks (Fig. 2 *D*) (WT; 0.36 ± 0.035, 0.4 ± 0.055. VEGF‐A‐KO; 0.54 ± 0.049, 0.78 ± 0.053. VEGF‐A‐KO × Neph‐VEGF_165_b; 0.23 ± 0.028, 0.6 ± 0.079, at 10 and 14 weeks, respectively, all units are mmHg^−1^ min^−1^) (*P* < 0.05; one‐way ANOVA).

### Structural studies

**Figure 3:**
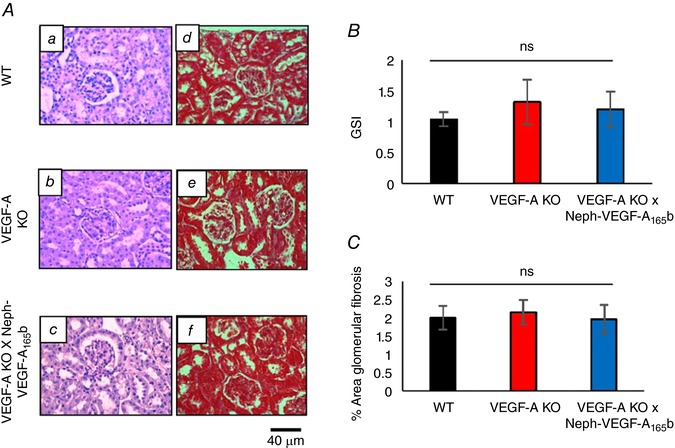
VEGF‐A‐KO mice do not develop glomerulosclerosis or fibrosis *A*, periodic acid‐Schiff (PAS) (*Aa*–*Ac*) and Masson's trichrome blue (*Ad*–*Af*) staining was performed to assess glomerulosclerosis and fibrosis. *B*, VEGF‐A‐KO mice did not develop any significant glomerulosclerosis (GSI) after 14 weeks (*n* = 3 mice, 25–30 glomeruli per mouse; *P* = not significant; one‐way ANOVA, Bonferroni correction for comparison between pairs). *C*, VEGF‐A‐KO mice also did not develop glomerular fibrosis after 14 weeks (*n* = 3 mice, 21–28 glomeruli per mouse; *P* = not significant; one‐way ANOVA, Bonferroni correction for comparison between pairs). No changes were observed in VEGF‐A‐KO × Neph‐VEGF‐A_165_b kidneys.

KD of podocyte VEGF‐A expression in VEGF‐A‐KO mice did not induce any glomerulosclerosis after 14 weeks, as assessed by the GSI scoring of PAS stained kidney cortex (Fig. 3 *A* and *B*). Furthermore, VEGF‐A‐KO mice did not develop any glomerular fibrosis (Fig. 3 *A* and *C*), as shown by Masson's trichrome staining of the kidney cortex. The over‐expression of podocyte VEGF‐A_165_b also had no effect on glomerulosclerosis and fibrosis in these mice. Therefore, the inducible KD of podocyte VEGF‐A did not result in a model of glomerulosclerosis in these mice.

**Figure 4:**
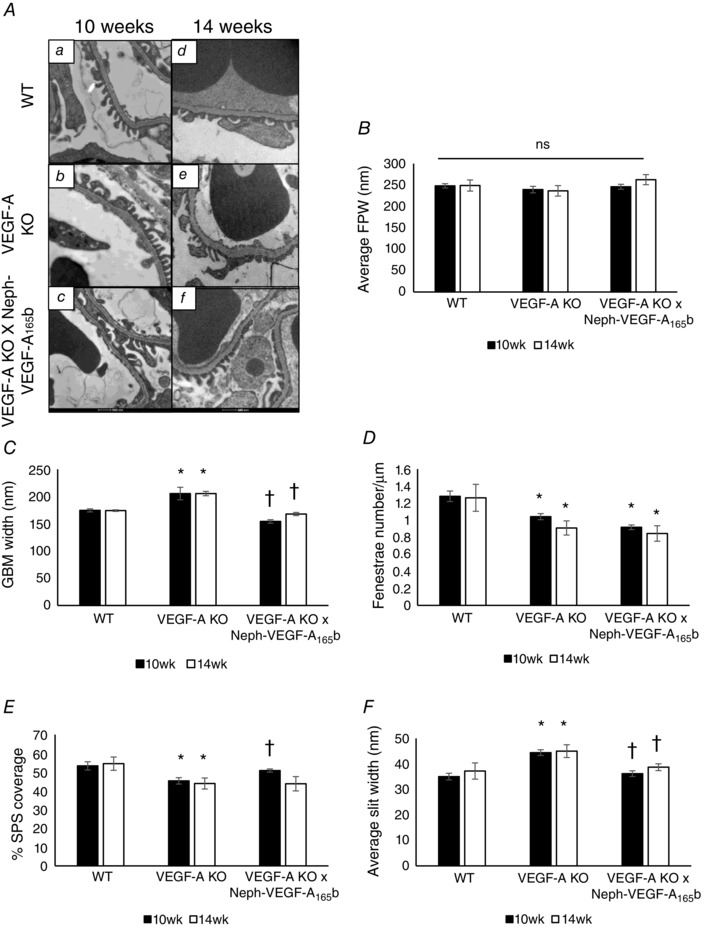
VEGF‐A‐KO mice develop several ultrastructural abnormalities, some of which are rescued in VEGF‐A‐KO × Neph‐VEGF‐A_165_b mice *A*, VEGF‐A‐KO mice did develop ultrastructural abnormalities (10 weeks; *Aa*–*Ac*, 14 weeks; *Ad*–*Ac*). Although there was no difference in foot process width at either time point (*P* > 0.05), (*B*) VEGF‐A‐KO mice develop thickening of the GBM after both 10 and 14 weeks of doxycycline (^*^ *P* < 0.05) compared to the control, which was fully rescued in the VEGF‐A‐KO × Neph‐VEGF‐A_165_b mouse (†*P* < 0.05) (*C*). VEGF‐A‐KO mice also had a significant reduction in endothelial fenestrae number per μm length of GBM (*D*), although there was no significant rescue in the VEGF‐A‐KO × Neph‐VEGF‐A_165_b mice at either time point (^*^ *P* < 0.05). *E*, VEGF‐A‐KO mice had a reduction in SPS coverage of the GBM compared to WT controls (^*^ *P* < 0.05), which was significantly rescued in the VEGF‐A‐KO × Neph‐VEGF‐A_165_b mice at 10 weeks but not 14 weeks (†*P* < 0.05). *F*, VEGF‐A‐KO mice had increased slit width between the podocyte foot processes (^*^ *P* < 0.05), which was fully rescued in the VEGF‐A‐KO × Neph‐VEGF‐A_165_b mice (†*P* < 0.05) (*n* = 3 mice; three gloms per mouse; one‐way ANOVA, Bonferroni correction for comparison between pairs).

Transmission electron microscopy analysis on samples collected after 10 and 14 weeks doxycycline induction revealed that the average podocyte foot process width did not change significantly across experimental groups (Fig. 4 *B*). However, VEGF‐A‐KO mice did develop an increased GBM width at both 10 and 14 weeks. Values returned to those observed in WT littermates when VEGF‐A_165_b was over‐expressed in the kidneys (WT; 174.06 ± 1.42 nm, VEGF‐A‐KO; 205.32 ± 4.02 nm, VEGF‐A‐KO × Neph‐VEGF_165_b; 167.8 ± 2.92 nm) (Fig. 4 *C*). The number of endothelial fenestrations was significantly decreased in both the VEGF‐A‐KO and VEGF‐A‐KO × Neph‐VEGF_165_b glomeruli (Fig. 4 *D*). The SPS coverage was significantly decreased in VEGF‐A‐KO glomeruli, and was rescued in VEGF‐A_165_b over‐expressors, only at 10 weeks (Fig. 4 *E*). VEGF‐A‐KO mice had an increased average slit width between the podocyte foot processes, which was fully rescued in VEGF‐A‐KO × Neph‐VEGF‐A_165_b mice at both 10 and 14 weeks (Fig. 4 *F*).

### Decreased PECAM‐1 expression and glomerular capillary circumference by VEGF‐A depletion is rescued by VEGF‐A_165_b over‐expression

**Figure 5:**
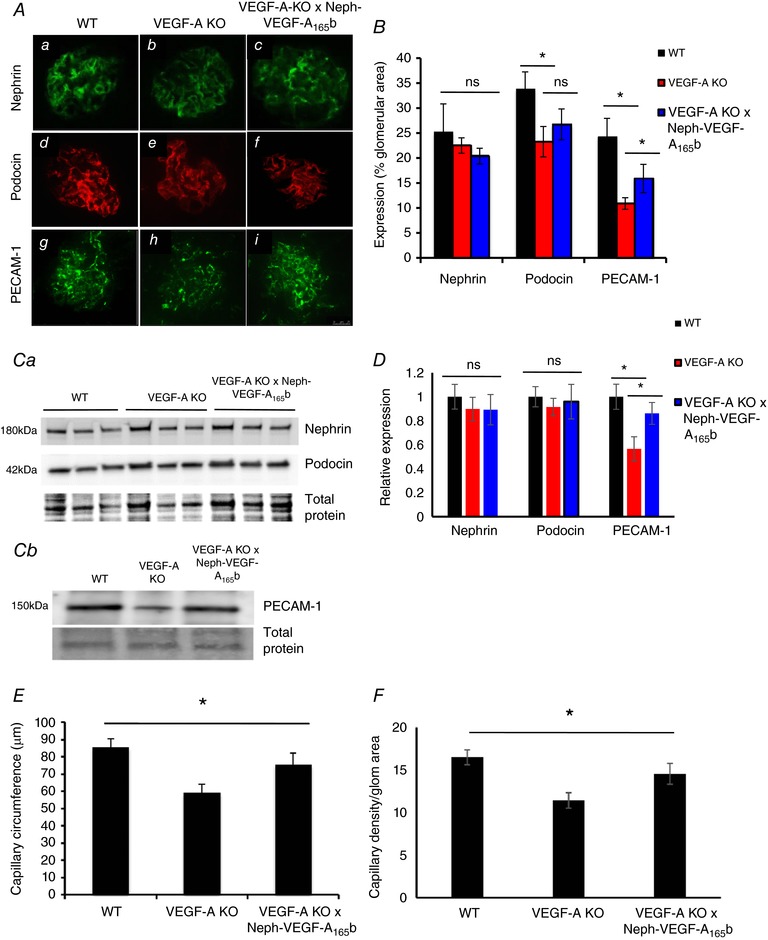
VEGF‐A‐KO mice have an altered endothelial phenotype, which is rescued in VEGF‐A‐KO × Neph‐VEGF_165_b mice *A*, sections from all three mice genotypes were stained for podocyte markers (podocin and nephrin) and endothelial marker (PECAM‐1). VEGF‐A‐KO mice had reduced glomerular podocin and PECAM‐1 staining, normalized to glomerular area, compared to WT controls, 14 weeks after induction with doxycycline. This was significantly rescued in the VEGF‐A‐KO × Neph‐VEGF‐A_165_b glomeruli. However, nephrin staining remained unchanged between groups (*n* = 3 mice, 36–48 glomeruli per mouse; ^*^ *P* < 0.05; one‐way ANOVA, Bonferroni correction for comparison between pairs). Quantification of the staining normalized to glomerular area is represented graphically in (*B*). *C* and *D*, western blotting showed only a change in the protein expression of PECAM‐1 occurred (*Cb*), with a reduction in expression found in VEGF‐A‐KO mice, which is rescued by podocyte over‐expression of VEGF‐A_165_b. Expression of the podocyte proteins nephrin and podocin remain unchanged (*Ca*), (*n* = 4 mice; ^*^ *P* < 0.05; one‐way ANOVA, Bonferroni correction for comparison between pairs). *E*, the capillary loop circumference was reduced in VEGF‐A‐KO glomeruli compared to WT controls, which was significantly rescued in the VEGF‐A‐KO × Neph‐VEGF‐A_165_b glomeruli (*n* = 3 mice, 36–50 glomeruli per mouse, two or three loops per glomerulus; ^*^ *P* < 0.05; one‐way ANOVA, Bonferroni correction for comparison between pairs). *F*, the capillary density normalized to glomerular area was reduced in VEGF‐A‐KO mice relative to WT controls, which was rescued in VEGF‐A‐KO × Neph‐VEGF‐A_165_b mice (*n* = 3 mice, 36–52 glomerular sections per mouse; ^*^ *P* < 0.05; one‐way ANOVA, Bonferroni correction for comparison between pairs).

To further investigate potential molecular mechanisms by which VEGF‐A_165_b is able to rescue the above‐described phenotype, we carried out immunofluorescence on kidney cortex sections for various podocyte and endothelial cell markers, and investigated their expression using western blotting. Using immunofluorescence, podocin and PECAM‐1 expression were both decreased in VEGF‐A‐KO mice at 14 weeks post‐induction. PECAM‐1 (although not podocin) expression was partially rescued in VEGF‐A‐KO × Neph‐VEGF‐A_165_b mice (Fig. 5 *A* and *B*). However, western blotting only detected a reduction in the expression of PECAM‐1 in the VEGF‐A‐KO kidney cortex, which was rescued in VEGF‐A‐KO × Neph‐VEGF‐A_165_b mice (Fig. 5 *C* and *D*). Nephrin expression was not changed across the littermate groups. Interestingly, capillary circumference and capillary density were also decreased in VEGF‐A‐KO glomeruli, and this was rescued by over‐expression of VEGF‐A_165_b in the podocytes (Fig. 5 *E* and *F*). Taken together, these results suggest that the protective effect of VEGF‐A_165_b *in vivo* is mediated via actions on the endothelial cell compartment.

### VEGF‐A_165_b increases expression of VEGFR2 in glomerular endothelial cells in VEGF‐A‐KO × Neph‐VEGF‐A_165_b mice

VEGF‐A_165_b is reported to signal through VEGFR2 activation in human umbilical vein endothelial cells (Cebe Suarez *et al*. 2006). We recently showed that, in Neph‐VEGF‐A_165_b mice, podocytic VEGF‐A_165_b induces both increased expression and phosphorylation of VEGFR2 in glomerular endothelial cells *in vivo* (Oltean *et al*. 2015). We therefore investigated whether a VEGF‐A_165_b ‐mediated rescue of VEGFR2 expression in VEGF‐A‐KO mice could account for the phenotypic rescue observed.

**Figure 6:**
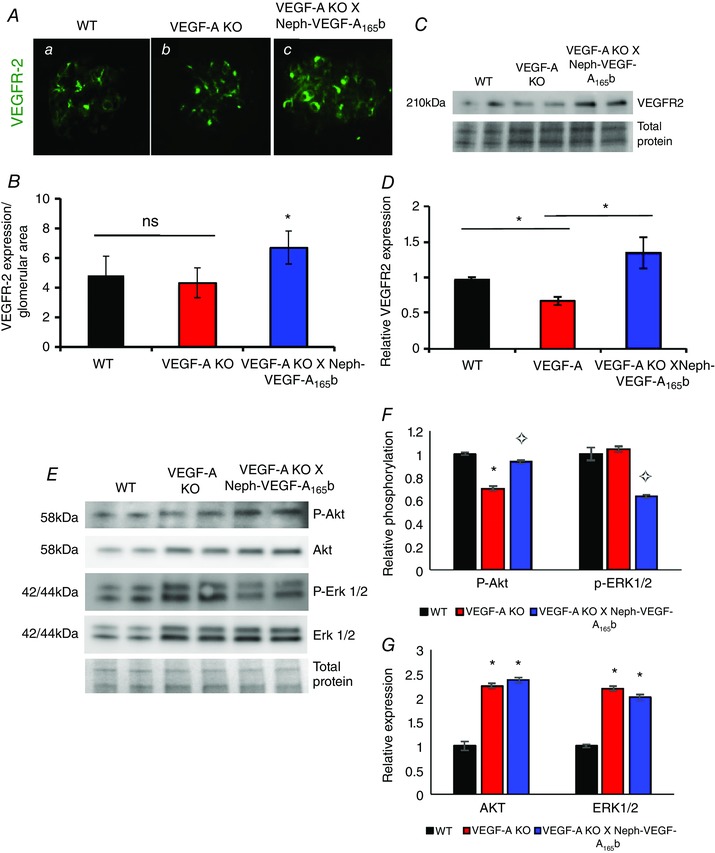
VEGF‐A_165_b may act through VEGFR2 in glomerular endothelial cells *in vivo* Glomerular VEGFR2 staining (green) (*A*), normalized to glomerular area, was increased in VEGF‐A‐KO × Neph‐VEGF‐A_165_b (c) glomeruli compared to WT (a) and VEGF‐A‐KO (b) controls (*B*) (*n* = 3 mice, 14–17 glomeruli, ^*^ *P* < 0.05; one‐way ANOVA, Bonferroni correction for comparison between pairs). *C*, protein from sieved glomeruli showed VEGFR2 expression to be reduced in VEGF‐A‐KO mice compared to WT controls. VEGF‐A‐KO × Neph‐VEGF‐A_165_b mice have rescued VEGFR2 glomerular protein expression, with the analysis shown in (*D*) (*n* = 5, ^*^ *P* ≤ 0.05; one‐way ANOVA, Bonferroni correction for comparison between pairs). *E*, VEGF‐A‐KO induces a reduction in the phosphorylation of Akt (Ser 453), which is reversed in the VEGF‐A‐KO × Neph‐VEGF‐A_165_b glomeruli (*F*). VEGF‐A‐KO induces no change in the phosphorylation of ERK1/2; however, VEGF‐A‐KO × Neph‐VEGF‐A_165_b glomeruli have a reduction in ERK1/2 phosphorylation relative to WT glomeruli (*E* and *F*). *G*, glomerular Akt and ERK1/2 expression is increased in both VEGF‐A‐KO and VEGF‐A‐KO × Neph‐VEGF‐A_165_b mice relative to WT controls (*n* = 4, ^*^ *P* < 0.05 WT *vs*. VEGF‐A‐KO, ✧*P* < 0.05 VEGF‐A‐KO *vs*. VEGF‐A‐KO × Neph‐VEGF‐A_165_b; one‐way ANOVA, Bonferroni correction for comparison between pairs).

Immunofluorescence staining and western blotting demonstrated that there was an up‐regulation of the receptor expression in VEGF‐A‐KO × Neph‐VEGF‐A_165_b glomeruli compared to VEGF‐A‐KO (Fig. 6 *A–D*). The increase in VEGFR2 demonstrates the protective effects of VEGF‐A_165_b on the glomerular endothelium. Furthermore, when investigating the effects of chronic VEGF‐A‐KO and over‐expression of VEGF‐A_165_b on the VEGF‐A signalling pathway, we found that a KO of VEGF‐A resulted in an up‐regulation in the glomerular expression of Akt and ERK1/2, both of which were also observed in VEGF‐A‐KO × Neph‐VEGF‐A_165_b glomeruli (Fig. 6 *E* and *G*). However, chronic VEGF‐A‐KO resulted in decreased activity of these kinases, as indicated by a reduction in the phosphorylation of Akt at Serine 453, as well as no change in the phosphorylation of ERK1/2. This was altered by the over‐expression of VEGF‐A_165_b, resulting in an increase in the phosphorylation of the cyto‐protective Akt, and a decrease in the phosphorylation of ERK1/2 (Fig. 6 *F*).

**Figure 7:**
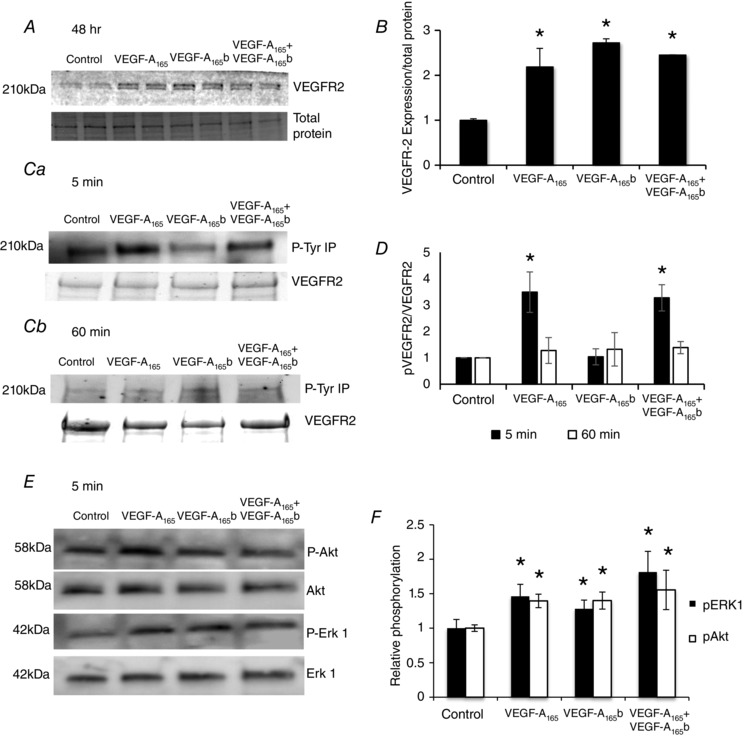
VEGF‐A_165_ and VEGF‐A_165_b increase the expression but have different effects on phosphorylation of VEGFR2 in ciGEnCs Differentiated ciGEnCs were treated with 1 nm VEGF‐A_165_, VEGF‐A_165_b, or a combination of both for 48 h (*A*) and expression of VEGFR2 measured by immunoblotting. *B*, VEGFR2 was increased in all treatments compared to the untreated control (*n* = 3, ^*^ *P* < 0.05, one‐way ANOVA, Bonferroni correction for comparison between pairs). No change in expression of VEGFR2 was observed at treatment times shorter than 48 h. *Ca* and *Cb*, treatment with VEGF‐A_165_, and a combination of VEGF‐A_165_ and VEGF‐A_165_b induced phosphorylation of VEGFR2 after 5 min, which was diminished after 1 h. However, no phosphorylation of the receptor was seen with treatment of VEGF‐A_165_b alone. *D*, phosphorylation of VEGFR2 was normalized to VEGFR2 expression, and then to control (*n* = 3, ^*^ *P* < 0.05, one‐way ANOVA, Bonferroni correction for comparison between pairs). *E*, both VEGF‐A isoforms resulted in an increase in the phosphorylation of ERK1 and Akt in GEnCs after 5 min of treatment. *F*, phosphorylation of ERK1 and Akt was normalized to total ERK1 and Akt expression, and then to control (*n* = 4 or 5, ^*^ *P* < 0.05, one‐way ANOVA, Bonferroni correction for comparison between pairs).

To confirm that VEGF‐A_165_b could act through VEGFR2 in ciGEnCs, as well as to understand the differences between the two splice isoforms, cells were treated with VEGF‐A_165_, VEGF‐A_165_b, or both. All treatments increased the expression of VEGFR2 at 48 h (Fig. 7 *A, B*), as observed *in vivo*. Phosphorylation of VEGFR2 was robustly increased by VEGF‐A_165_ and a combination treatment of VEGF‐A_165_ and VEGF‐A_165_b at 5 min, which was diminished 1 h later. However, VEGF‐A_165_b treatment alone did not induce VEGFR2 phosphorylation at either time point (Fig. 7 *C* and *D*). When looking downstream of VEGFR2 activation, both isoforms of VEGF‐A result in an increase in the phosphorylation of p42 MAPK (ERK1) and Akt after 5 min of treatment in ciGEnCs (Fig. 7 *E* and *F*).

### VEGF‐A_165_b differentially regulates a set of genes in GEnCs compared to VEGF‐A_165_

**Figure 8:**
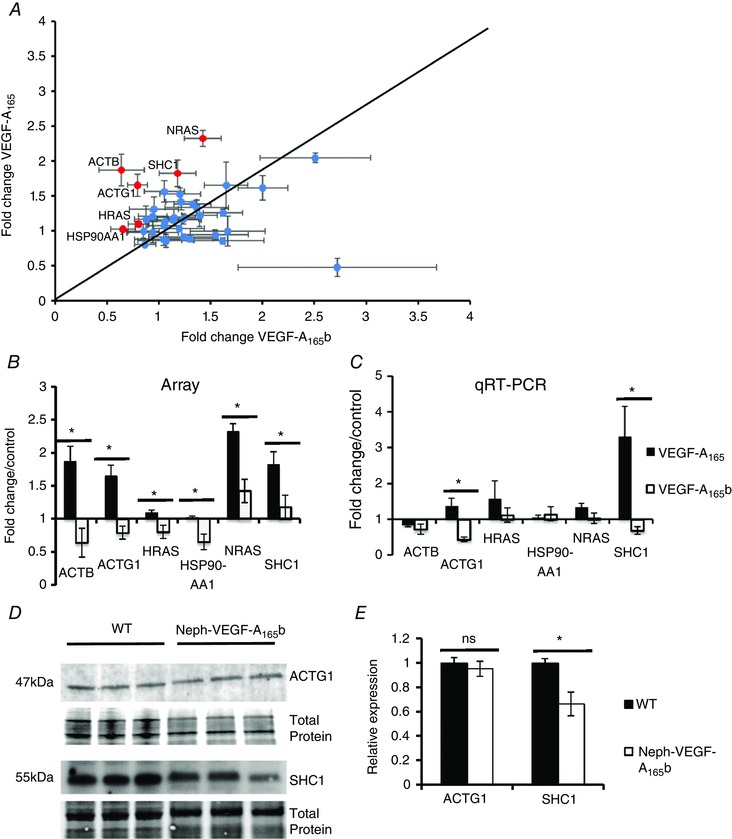
VEGF‐A_165_b differentially regulates the expression of several candidate genes in the human VEGF‐A signalling pathway in ciGEnCs **A**, a TaqMan Fast PCR Array for the the human VEGF‐A pathway highlighted six candidate genes (*B*) that were significantly down‐regulated by 1 h of treatment with VEGF‐A_165_b compared to VEGF‐A_165_ in ciGEnCs; ACTB, ACTG1, HRAS, HSP90AA1, NRAS and SHC1 (*n* = 4, ^*^ *P* < 0.05, Student's *t* test for comparison between groups). *C*, quantitative RT‐PCR validation of the six candidate genes using gene specific primers confirmed that two of the genes were significantly down‐regulated by 1 h of treatment with VEGF‐A_165_b compared to VEGF‐A_165_: ACTG1 and SHC1 (*n* = 4, ^*^ *P* < 0.05, Student's *t* test for comparison between groups). *D*, up‐regulation of SHC1 (but not ACTG1) protein expression in the kidney cortex was observed in Neph‐VEGF‐A_165_b over‐expressing mice. Protein expression shown relative to WT control expression (*E*) (*n* = 3, ^*^ *P* < 0.05, Student's *t* test for comparison between groups).

To further understand the molecular pathways by which VEGF‐A_165_b mediates its effects in the glomerulus compared to VEGF‐A_165,_ we used a custom‐designed quantitative RT‐PCR array specific for the VEGF‐A signalling axis (TaqMan Fast Human VEGF‐A PCR array; Applied Biosciences; 44 candidate genes plus four control genes). Six genes were differentially expressed during VEGF‐A_165_b treatment compared to VEGF‐A_165_ (Fig. 8 *A* and *B*). ACTG1 and SHC1 are involved in endothelial cell migration, mitogenesis and apoptosis, and were further validated by quantitative RT‐PCR (Fig. 8 *C*). Their expression is increased by VEGF‐A_165_ but decreased by VEGF‐A_165_b, providing a possible explanation for the protective effects of VEGF‐A_165_b on glomerular vasculature. To confirm these findings *in vivo*, we first analysed the protein expression of the two genes in kidney cortex extracts from Neph‐VEGF‐A_165_b over‐expressors compared to WT littermates. Although ACTG1 is not changed (it is possibly easier to see a difference in glomerular extracts), there is a decrease in SHC1 in Neph‐VEGF‐A_165_b over‐expressors (Fig. 8 *D* and *E*).

## Discussion

Despite progress made in recent years, CKD remains a major health challenge worldwide not only because of the immense burden of end‐stage renal disease and chronic dialysis, but also as a result of systemic cardiovascular complications and associated morbidities (Matsushita *et al*. 2010). Therefore, it is critical to further our understanding of the molecular mechanisms disrupted during CKD progression, as well as to find ways of manipulating them for therapeutic benefit. Without doubt, CKD is multifactorial and extremely complex in its pathogenesis; we can certainly progress our understanding by studying individual genes that have been shown to be tightly associated with CKD progression. One such gene is VEGF‐A, whose involvement in different renal pathologies has been studied for over 20 years, and it was recently shown to correlate with the estimated GFR in patients with CKD (Martini *et al*. 2014). This is the first study to assess the effects of podocyte‐specific VEGF‐A_165_b over‐expression in a model of podocyte depletion of other isoforms of VEGF‐A. To study the specific effect of only VEGF‐A and its splice isoforms on kidney function, we used an inducible, podocyte‐specific VEGF‐A‐KO mouse where the VEGF‐A_165_b isoform could be over‐expressed in a constitutive manner. The advantage of using this model compared to other widely used chronic kidney disease models, such as the 5/6 nephrectomy and unilateral ureteric obstruction models, is that we can look specifically at the effect of VEGF‐A expression and splicing on kidney function.

In our mice, VEGF‐A levels were progressively depleted from the kidney to ∼40% at 14 weeks post‐induction with doxycycline. This was the case in both the VEGF‐A‐KO and the VEGF‐A‐KO × Neph‐VEGF‐A_165_b model with respect to VEGF‐A_164_, with the exception that the VEGF‐A‐KO × Neph‐VEGF‐A_165_b mouse over‐expressed specifically the VEGF‐A_165_b isoform in the podocytes. This over‐expression did not have an effect on the depletion of total mouse VEGF‐A. The VEGF‐A‐KO did not result in a dramatic deterioration in kidney function, as described previously in some studies (Eremina & Quaggin, 2004; Eremina *et al*. 2008). This is not unexpected because we only target one gene in what is a very complex and multifactorial system of normal, mature glomerular function. We instead develop a model of microalbuminuria and increased glomerular permeability as a result of ultrastructural glomerular changes in response to a progressive KD of podocyte VEGF‐A expression. In our mice, KO was induced over a 10–14 week period, once the mice had reached maturity (> 8 weeks of age). In previous studies, KO was induced during pre‐ or postnatal development (3–3.5 weeks) (Eremina & Quaggin, 2004; Sivaskandarajah *et al*. 2012) when the glomerulus is still forming, which results in a more severe phenotype as a result of the important role that VEGF‐A plays in glomerular development (Eremina & Quaggin, 2004). Interestingly, small interfering RNA KD also caused a more severe phenotype (Veron *et al*. 2012), even when induced in adult mice. Differences might also be related to strain/genetic background, animal facility or the presence of associated confounding factors. Our mice were generated on a C57BL/6 background because many genetically modified mice are a result of the advantages of this strain (Seong *et al*. 2004), which is now widely accepted to be resistant to severe albuminuria and glomerulosclerosis (Zheng *et al*. 1998). In our mice, we observed an increase in proteinuria at 10 and 14 weeks post‐induction with doxycycline; thus, we obtained a model that mimics the correlation between decreased levels of VEGF‐A and CKD progression seen in humans with respect to proteinuria (Martini *et al*. 2014). Over‐expression of VEGF‐A_165_b is able to rescue proteinuria, as well as glomerular water permeability, a sensitive measure of kidney function (Salmon *et al*. 2006). At 14 weeks, there is no full rescue of the glomerular water permeability by over‐expression of VEGF‐A_165_b, as seen at 10 weeks, which is probably a result of the further depletion of podocyte VEGF‐A expression between these time points (40–60% depletion) giving rise to a more severe phenotype (Figs 1 and 2). We would have expected the rescue of the uACR by VEGF‐A_165_b at 14 weeks to be reflected in the glomerular water permeability measurements at the same time point, in the same mice. However, as previous studies using this technique have demonstrated (Oltean *et al*. 2012; Oltean *et al*. 2015), this is not always the case. The glomerular water permeability assay is a more sensitive technique and thus is representative of individual glomeruli, whereas the uACR measurements are reflective of the whole kidney function and can be less sensitive. Furthermore, the assays measure two different factors (i.e. water and albumin)n under different conditions; therefore, small differences in the measurements are expected and this explains why we carry out both techniques on all mice. Because of the increased depletion of podocyte VEGF‐A observed at 14 weeks compared to 10 weeks, we decided to focus much of our analysis on this later time point.

No major structural abnormalities such as glomerulosclerosis and fibrosis were found in this model (Fig. 3), which explains why we only observe a mild phenotype with progressive depletion of podocyte VEGF‐A. Ultrastructural (electron microscopy) studies found moderate alterations in glomerular structure (Fig. 4), including a modest increase in GBM thickness, as observed in another published model of podocyte VEGF‐A‐KO (Eremina *et al*. 2008). Alterations to the podocytes included an increase in the slit width, indicating the beginnings of foot process effacement. This was rescued in VEGF‐A‐KO × NephVEGF‐A_165_b mice. Nephrin expression is not changed across experimental groups (Fig. 5), suggesting that the slit width modifications may be a result of the rearrangement of nephrin (or other slit proteins) bonds. Interestingly, we also observed a decrease in SPS coverage similar to that observed when VEGF‐A_164_ is over‐expressed in the kidney in a previously published transgenic model (Oltean *et al*. 2012). As observed in the VEGF‐A_164_ over‐expression model, SPS coverage is rescued by VEGF‐A_165_b in the model used in the present study, suggesting that the differences in the ways the two splice isoforms interact with their receptors and the consequent functions depend on their absolute abundance. Therefore, it appears that one of the mechanisms by which VEGF‐A_165_b is acting in the kidney to reduce glomerular water permeability under pathological conditions is by maintenance of SPS coverage. Ultrastructural alterations to the glomerular endothelial cells, where VEGF‐A signalling occurs, included a reduction in fenestrae number in VEGF‐A‐KO mice. VEGF‐A is critical for production and maintenance of endothelial fenestrae because a reduction in VEGF‐A signalling has previously shown a loss of the normal fenestrated phenotype (Oltean *et al*. 2015) and treatment of ciGEnCs with VEGF‐A has been shown to induce fenestrations *in vitro* (Satchell *et al*. 2006). The reduction in endothelial fenestration number remained decreased in VEGF‐A‐KO × NephVEGF‐A_165_b mice, which is probably a result of the previously described effect that VEGF‐A_165_b has on endothelial fenestrae closure (Qiu *et al*. 2010).

Although we did not obtain a full KO of VEGF‐A from the podocytes, the evidence presented suggests that over‐expression of VEGF‐A_165_b is not only beneficial to the kidney, but also appears to be sufficient to maintain glomerular function in this model. How is the action of VEGF‐A_165_b mediated in the kidney and how is it different to that of VEGF‐A_165_? Staining with podocyte and endothelial specific markers indicated that the rescue effect of VEGF‐A_165_b is mediated through restoring endothelial cell expression, as demonstrated by an increase in PECAM‐1 and VEGFR2, and glomerular capillary circumference (Fig. 5). Although we did not observe any changes in the expression of VEGFR2 using immunofluorescence, we consider this to be a result of the technique itself; assessment of protein expression with immunofluorescence can give variable results depending on the area of the glomerulus being sectioned. Western blotting is a more accepted method for the quantification of protein expression. We also cannot rule out tubular contamination in the sieved glomeruli protein extract used in the western blotting, which could indicate that podocyte depletion of VEGF‐A could induce tubular changes in these mice. Future experiments could investigate this. VEGF‐A_165_b increases the glomerular expression of VEGFR2 *in vivo* (Fig. 6), as previously described by Oltean *et al*. (2015), and appears to be acting through VEGFR2 in the glomerulus, as demonstrated by the decrease in glomerular water permeability in the presence of tyrosine kinase inhibitors, although other receptors cannot be fully ruled out. Downstream of VEGFR2, a KO of VEGF‐A and over‐expression of VEGF‐A_165_b both increased the expression of the kinases Akt and ERK1/2. However, the kinase activity of Akt is reduced in VEGF‐A‐KO glomeruli, probably as a result of a lack of VEGFR signalling. This is reversed by VEGF‐A_165_b over‐expression, suggesting that VEGF‐A_165_b is signalling via a VEGFR that has cyto‐protective effects, such as reduced endothelial cell apoptosis, restoring normal glomerular function. Although chronic exposure to VEGF‐A_165_b increases the phosphorylation of Akt, it had the opposite effect on the phosphorylation of another kinase; ERK1. Activation of ERK1 was reduced, which is reflective of previous studies reporting that VEGF‐A_165_b rapidly inactivates VEGFR2 phosphorylation, therefore resulting in a weak, transient phosphorylation of downstream targets such as ERK1/2 (Kawamura *et al*. 2008). Interestingly, the same study reported a similar effect on the phosphorylation of Akt in response to VEGF‐A_165_b, thereby providing further evidence for the possibility of VEGF‐A_165_b acting through other receptors in the glomerulus.

This is the first study to assess the effects of VEGF‐A_165_b treatment of ciGEnCs in culture, providing further mechanistic evidence of the role played by VEGF‐A_165_b in the glomerulus. The increase in expression of VEGFR2 can be reproduced *in vitro* by treating ciGEnCs with VEGF‐A_165_b (Fig. 7). Although VEGF‐A_165_b did not induce phosphorylation of VEGFR2, it did result in the initial phosphorylation of ERK1 and Akt, as previously reported in human microvascular endothelial cells (Kawamura *et al*. 2008), both of which are pro‐survival factors in endothelial cells. Once again, this could indicate that, although inhibitory to VEGFR2, VEGF‐A_165_b may be signalling via other receptors in GEnCs, of which are yet to be determined.

A VEGF‐A pathway PCR array revealed decreased expression of ACTG1 and SHC1 mRNA when ciGEnCs were stimulated with VEGF‐A_165_b compared to VEGF‐A_165_ (Fig. 8). When looking at the expression of these two proteins in the renal cortex of mice that over‐express VEGF‐A_165_b in the podocytes, we see a down‐regulation of SHC1, although not ACTG1 expression, mimicking that seed in culture with regard to SHC1. These genes are involved in migration and proliferation of endothelial cells in response to VEGF‐A signalling (Cross *et al*. 2003; Oshikawa *et al*. 2012) and the down‐regulation of SHC1 provides some insight into the mechanism of action of VEGF‐A_165_b under normal physiological conditions. Interestingly, a KO of SHC1 has been shown to be protective in a model of albuminuria (Menini *et al*. 2006). However, when there is a KD of VEGF‐A in the glomeruli, VEGF‐A_165_b over‐expression results in the opposite effect on ACTG1 and SHC1, no longer down‐regulating the expression of the two proteins. Therefore, it is apparent that a change in the balance of the two isoform families can alter the downstream signalling pathways that are activated. This also gives increasing strength to the hypothesis that VEGF‐A_165_b is acting on other receptors, such as VEGF receptor 1 and platelet derived growth factor receptor (Cumpanas *et al*. 2016), or is resulting in a transient weak phosphorylation of VEGFR2, as described previously, which we were unable to detect in our GEnCs (Kawamura *et al*. 2008). Further investigations are required to clarify the complex pathways involved in the VEGF‐A_165_b mechanistic action. However, it is evident that the ratio of VEGF‐A_xxx_/VEGF‐A_xxx_b expression determines the downstream pathways activated/inhibited.

In conclusion, the present study demonstrates that the VEGF‐A_165_b splice isoform is sufficient to support kidney function in a podocyte specific VEGF‐A‐KO model of microalbuminuria, and may explain why treatments using bevacizumab, an antibody against all forms of VEGF, are deleterious to the kidney (Eremina *et al*. 2008). In a recent study, we reported that patients with early stage diabetic nephropathy appear to up‐regulate VEGF‐A_165_b isoform expression in the kidney as a compensatory mechanism; however, this is lost with disease progression (Oltean *et al*. 2015). Together with the findings of the present study, we have gained further evidence for the importance of the VEGF‐A splicing ratio in kidney function. The present study has enabled us to determine the protective effects of VEGF‐A_165_b in a model when all other VEGF‐A isoforms are depleted, and novel mechanistic evidence is provided regarding the importance of the VEGF‐A splicing ratio in kidney function, specifically in glomerular endothelial cells. Therefore, there is a compelling case for investigating the therapeutic avenues that aim to switch splicing towards VEGF‐A_165_b in the kidney with the potential to slow the progression of CKD via the protection of kidney function.

## Additional information

### Competing interests

The authors declare that they have no competing financial interests.

### Author contributions

SO, DB, SH and AHJS designed the research. MS and CRN performed the research. MS and SO analysed the data. MS and SO wrote the paper, which was critically reviewed by the other authors. All authors have approved submission of the revised version. All authors agree to be accountable for all aspects of the work. All persons designated as authors qualify' for authorship, and all those who qualify for authorship are listed.

### Funding

Funding for the present study was provided by BBSRC (BB/J007293/2), the Medical Research Council (G10002073), British Heart Foundation (PG/15/53/31371) and Richard Bright VEGF Research Trust.

**Translational perspectives**

Chronic kidney disease (CKD) is a major health problem that affects ∼15% of people in the Western world. A large proportion of these patients progress to end‐stage renal failure and dialysis, resulting in increasing costs in healthcare. It is therefore very important to further understand CKD disease mechanisms and to develop novel therapeutic approaches. Vascular endothelial growth factor A (VEGF‐A) is essential in maintaining kidney homeostasis, with both increases and decreases in expression being associated with kidney malfunction. There is a strong association of decreased VEGF‐A levels with CKD. The splice isoform VEGF‐A_165_b has been previously shown to be renoprotective. In the present study, we demonstrate, in a mouse model of microalbuminuria with progressive depletion of all VEGF‐A isoforms from the kidney, that over‐expression of the VEGF‐A_165_b isoform alone rescues the phenotype. This suggests that modulation of VEGF‐A splicing may be a viable therapeutic avenue for CKD.

## References

[tjp12462-bib-0001] Allison SJ (2010). Meta‐analysis confirms relationship between eGFR, albuminuria and risk of mortality. Nat Rev Nephrol 6, 501. 20815092 10.1038/nrneph.2010.105

[tjp12462-bib-0002] Bates DO , Cui TG , Doughty JM , Winkler M , Sugiono M , Shields JD , Peat D , Gillatt D & Harper SJ (2002). VEGF165b, an inhibitory splice variant of vascular endothelial growth factor, is down‐regulated in renal cell carcinoma. Cancer Res 62, 4123–4131. 12124351

[tjp12462-bib-0003] Bevan HS , van den Akker NM , Qiu Y , Polman JA , Foster RR , Yem J , Nishikawa A , Satchell SC , Harper SJ , Gittenberger‐de Groot AC & Bates DO (2008). The alternatively spliced anti‐angiogenic family of VEGF isoforms VEGF(xxx)b in human kidney development. Nephron Physiol 110, 57–67. 10.1159/000177614 PMC2635558 19039247

[tjp12462-bib-0004] Cebe Suarez S , Pieren M , Cariolato L , Arn S , Hoffmann U , Bogucki A , Manlius C , Wood J & Ballmer‐Hofer K (2006). A VEGF‐A splice variant defective for heparan sulfate and neuropilin‐1 binding shows attenuated signaling through VEGFR‐2. Cell Mol Life Sci 63, 2067–2077. 16909199 10.1007/s00018-006-6254-9 PMC11136335

[tjp12462-bib-0005] Chen HX & Cleck JN (2009). Adverse effects of anticancer agents that target the VEGF pathway. Nat Rev Clin Oncol 6, 465–477. 19581909 10.1038/nrclinonc.2009.94

[tjp12462-bib-0006] Cross MJ , Dixelius J , Matsumoto T & Claesson‐Welsh L (2003). VEGF‐receptor signal transduction. Trends Biochem Sci 28, 488–494. 13678960 10.1016/S0968-0004(03)00193-2

[tjp12462-bib-0007] Cumpanas, AA , Cimpean, AM , Ferician, O , Ceausu, RA , Sarb, S , Barbos, V , Dema, A , and Raica, M (2016). The involvement of PDGF‐B/PDGFRβ axis in the resistance to antiangiogenic and antivascular therapy in renal cancer. Anticancer Res 36, 2291–2295. 27127135

[tjp12462-bib-0008] Eremina V , Jefferson JA , Kowalewska J , Hochster H , Haas M , Weisstuch J , Richardson C , Kopp JB , Kabir MG , Backx PH , Gerber HP , Ferrara N , Barisoni L , Alpers CE & Quaggin SE (2008). VEGF inhibition and renal thrombotic microangiopathy. N Engl J Med 358, 1129–1136. 18337603 10.1056/NEJMoa0707330 PMC3030578

[tjp12462-bib-0009] Eremina V & Quaggin SE (2004). The role of VEGF‐A in glomerular development and function. Curr Opin Nephrol Hypertens 13, 9–15. 15090854 10.1097/00041552-200401000-00002

[tjp12462-bib-0010] Go AS , Chertow GM , Fan D , McCulloch CE & Hsu CY (2004). Chronic kidney disease and the risks of death, cardiovascular events, and hospitalization. N Engl J Med 351, 1296–1305. 15385656 10.1056/NEJMoa041031

[tjp12462-bib-0011] Harper SJ & Bates DO (2008). VEGF‐A splicing: the key to anti‐angiogenic therapeutics? Nat Rev Cancer 8, 880–887. 18923433 10.1038/nrc2505 PMC2613352

[tjp12462-bib-0012] Kawamura H , Li X , Harper SJ , Bates DO & Claesson‐Welsh L (2008). Vascular endothelial growth factor (VEGF)‐A165b is a weak in vitro agonist for VEGF receptor‐2 due to lack of coreceptor binding and deficient regulation of kinase activity. Cancer Res 68, 4683–4692. 18559514 10.1158/0008-5472.CAN-07-6577

[tjp12462-bib-0013] Martini S , Nair V , Keller BJ , Eichinger F , Hawkins JJ , Randolph A , Boger CA , Gadegbeku CA , Fox CS , Cohen CD , Kretzler M , European Renal c DNAB , Cohort CP & Consortium CK (2014). Integrative biology identifies shared transcriptional networks in CKD. J Am Soc Nephrol 25, 2559–2572. 24925724 10.1681/ASN.2013080906 PMC4214523

[tjp12462-bib-0014] Matsushita K , van der Velde M , Astor BC , Woodward M , Levey AS , de Jong PE , Coresh J & Gansevoort RT (2010). Association of estimated glomerular filtration rate and albuminuria with all‐cause and cardiovascular mortality in general population cohorts: a collaborative meta‐analysis. Lancet 375, 2073–2081. 20483451 10.1016/S0140-6736(10)60674-5 PMC3993088

[tjp12462-bib-0015] Meguid El Nahas A & Bello AK (2005). Chronic kidney disease: the global challenge. Lancet 365, 331–340. 15664230 10.1016/S0140-6736(05)17789-7

[tjp12462-bib-0016] Menini S , Amadio L , Oddi G , Ricci C , Pesce C , Pugliese F , Giogio M , Migliaccio E , Pelicci P , Iacobini C , & Pugliese G (2006). Deletion of P66Shc longevity gene protects against experimental diabetic glomerulopathy by preventing diabetes‐induced oxidative stress. Diabetes 55, 1642–1650. 16731826 10.2337/db05-1477

[tjp12462-bib-0017] Ollero M & Sahali D (2014). Inhibition of the VEGF signalling pathway and glomerular disorders. Nephrol Dial Transplant 9, 1449–55 10.1093/ndt/gfu368 25480873

[tjp12462-bib-0018] Oltean S , Neal CR , Mavrou A , Patel P , Ahad T , Alsop C , Lee T , Sison K , Qiu Y , Harper SJ , Bates DO & Salmon AH (2012). VEGF165b overexpression restores normal glomerular water permeability in VEGF164‐overexpressing adult mice. Am J Physiol Renal Physiol 303, F1026–F1036. 22811490 10.1152/ajprenal.00410.2011 PMC3469682

[tjp12462-bib-0019] Oltean S , Qiu Y , Ferguson JK , Stevens M , Neal C , Russell A , Kaura A , Arkill KP , Harris K , Symonds C , Lacey K , Wijeyaratne L , Gammons M , Wylie E , Hulse RP , Alsop C , Cope G , Damodaran G , Betteridge KB , Ramnath R , Satchell SC , Foster RR , Ballmer‐Hofer K , Donaldson LF , Barratt J , Baelde HJ , Harper SJ , Bates DO & Salmon AH (2015). Vascular endothelial growth factor‐A165b is protective and restores endothelial glycocalyx in diabetic nephropathy. J Am Soc Nephrol 26; 1889–1904. 25542969 10.1681/ASN.2014040350 PMC4520162

[tjp12462-bib-0020] Oshikawa J , Kim SJ , Furuta E , Caliceti C , Chen GF , McKinney RD , Kuhr F , Levitan I , Fukai T & Ushio‐Fukai M (2012). Novel role of p66Shc in ROS‐dependent VEGF signaling and angiogenesis in endothelial cells. Am J Physiol Heart Circ Physiol 302, H724–H732. 22101521 10.1152/ajpheart.00739.2011 PMC3353779

[tjp12462-bib-0021] Qiu Y , Ferguson J , Oltean S , Neal CR , Kaura A , Bevan H , Wood E , Sage LM , Lanati S , Nowak DG , Salmon AH , Bates D & Harper SJ (2010). Overexpression of VEGF165b in podocytes reduces glomerular permeability. J Am Soc Nephrol 21, 1498–1509. 20688932 10.1681/ASN.2009060617 PMC3013528

[tjp12462-bib-0022] Salmon AH , Neal CR , Bates DO & Harper SJ (2006). Vascular endothelial growth factor increases the ultrafiltration coefficient in isolated intact Wistar rat glomeruli. J Physiol 570, 141–156. 16239266 10.1113/jphysiol.2005.099184 PMC1464281

[tjp12462-bib-0023] Satchell SC , Tasman CH , Singh A , Ni L , Geelen J , von Ruhland CJ , O'Hare MJ , Saleem MA , van den Heuvel LP & Mathieson PW (2006). Conditionally immortalized human glomerular endothelial cells expressing fenestrations in response to VEGF. Kidney Int 69, 1633–1640. 16557232 10.1038/sj.ki.5000277

[tjp12462-bib-0024] Schrijvers BF , Flyvbjerg A & De Vriese AS (2004). The role of vascular endothelial growth factor (VEGF) in renal pathophysiology. Kidney Int 65, 2003–2017. 15149314 10.1111/j.1523-1755.2004.00621.x

[tjp12462-bib-0025] Seong E , Saunders TL , Stewart CL & Burmeister M (2004). To knockout in 129 or in C57BL/6: that is the question. Trends Genet 2, 59–62. 10.1016/j.tig.2003.12.006 14746984

[tjp12462-bib-0026] Sivaskandarajah GA , Jeansson M , Maezawa Y , Eremina V , Baelde HJ & Quaggin SE (2012). Vegfa protects the glomerular microvasculature in diabetes. Diabetes 61, 2958–2966. 23093658 10.2337/db11-1655 PMC3478549

[tjp12462-bib-0027] Snyder JJ , Foley RN & Collins AJ (2009). Prevalence of CKD in the United States: a sensitivity analysis using the National Health and Nutrition Examination Survey (NHANES) 1999–2004. Am J Kidney Dis 53, 218–228. 18950914 10.1053/j.ajkd.2008.07.034 PMC2664624

[tjp12462-bib-0028] Veron D , Villegas G , Aggarwal PK , Bertuccio C , Jimenez J , Velazquez H , Reidy K , Abrahamson DR , Moeckel G , Kashgarian M & Tufro A (2012). Acute podocyte vascular endothelial growth factor (VEGF‐A) knockdown disrupts alphaVbeta3 integrin signaling in the glomerulus. PLoS ONE 7, e40589. 22808199 10.1371/journal.pone.0040589 PMC3396653

[tjp12462-bib-0029] Zheng F , Striker GE , Esposito C , Lupia E & Striker LJ (1998). Strain differences rather than hyperglycemia determine the severity of glomerulosclerosis in mice. Kidney Int 54, 1999–2007. 9853264 10.1046/j.1523-1755.1998.00219.x

